# Prediction of disorders with significant coronary lesions using machine learning in patients admitted with chest symptom

**DOI:** 10.1371/journal.pone.0274416

**Published:** 2022-10-10

**Authors:** Jae Young Choi, Jae Hoon Lee, Yuri Choi, YunKyong Hyon, Yong Hwan Kim

**Affiliations:** 1 Department of Emergency Medicine, Inje University College of Medicine, Busan, Korea; 2 Department of Emergency Medicine, Dong-A University College of Medicine, Busan, Korea; 3 Division of Medical Mathematics, National Institute for Mathematical Sciences, Daejeon, Korea; 4 Department of Emergency Medicine, Samsung Changwon Hospital, Sungkyunkwan University School of Medicine, Changwon, Korea; Azienda Ospedaliero Universitaria Careggi, ITALY

## Abstract

**Background:**

The early prediction of significant coronary artery lesion, including coronary vasospasm, have yet to be studied. It is essential to discern the disorders with significant coronary lesions (SCDs) requiring coronary angiography from mimicking disease. We aimed to determine which of all clinical variables were more important using conventional logistic regression (cLR) and machine learning (ML).

**Materials:**

Of 3382 patients with chest pain/discomfort or dyspnea in whom CAG was performed, 1893 were included. All clinical data were divided as follows (i): Demographics, history, and physical examination; (ii): (i) plus electrocardiography; and (iii): (ii) plus echocardiography, and analyzed by cLR and ML.

**Results:**

In multivariable analysis via cLR, the AUC and accuracy of the model using the final 20 variables were 0.795 and 72.62%, respectively. In multivariable analysis via ML, the best AUCs in the internal validation were 0.8 with (i), 0.81 with (ii), 0.83 with (iii), and in external validation, the best AUCs were 0.71 with (i), 0.74 with (ii), and 0.79 with (iii). The best AUCs and accuracy of the fittest model including 21 importance variables by ML were 0.81 and 72.48% in internal validation; and 0.75 and 70.5% in external validation, respectively. The importance variables in ML and cLR were similar, but slightly different and the additional discriminators via ML were found.

**Conclusion:**

The assessment using the fittest importance variables can assist physicians in differentiating mimicking diseases in which coronary angiography may not be required in patients suspected of having acute coronary syndrome in emergency department.

## Introduction

Seventy-one percent of patients visiting the emergency room (ER) have chest pain [1], and 34.5% of them are diagnosed with acute coronary syndrome (ACS) [2]. However, it is not easy to clinically differentiate the disorders with significant coronary lesions (SCDs) for coronary angiography (CAG), including coronary artery disease (CAD), coronary spasm, coronary dissection, and coronary thrombus/embolus [3], in non-ST elevation ACS patients, from non-SCD [4]. It may be essential to access the probability of SCDs in the early stage before further evaluation.

Various diseases can mimic SCDs; furthermore, it takes a long time to perform the necessary examinations, such as coronary CT, stress cardiac MRI, treadmill test, stress echo, and stress radionuclide imaging, on potential SCD patients [5]. By performing basic history taking, blood tests, electrocardiography (ECG) and echocardiography on ER patients with chest pain/discomfort or dyspnea, it is possible to narrow down the range of suspected diseases, but it is still challenging to distinguish SCDs from several mimicking diseases that may have the same symptoms as SCDs, such as chest pain/discomfort or dyspnea, ECG change, and cardiac enzyme elevation [3].

When patients are suspected of having ACS, management such as immediate angiography and early or delayed invasive strategies, is scheduled and performed according to the prognosis of patients using risk stratification [6]. However, the diagnostic probability of SCDs in non-ST elevation ACS patients should be screened before designing the invasive treatment strategy according to the prognosis of the patients. The likelihood of ACS which was confirmed using CAG has been evaluated [7,8], but it is questionable whether the studies included vasospasm or severe mimicking diseases. Additionally, although pre-test probabilities of significant CAD were assessed in patients with non-anginal pain and dyspnea [9], most studies did not include patients with atypical chest discomfort or dyspnea as the rate of nonobstructive CAD was only 9.1% of non-ST elevation ACS [4].

There have been many studies on the diagnosis of CAD using machine learning (ML) and deep learning [10–15]. However, the results from these studies show discrepancies from the real-world clinical situation because these studies included patients who had only chest pain, excluded coronary spasm patients and mild or serious cases (even compared with healthy population), and missed valuable clinical data such as ECG or troponin I.

The aim of this study was to determine the best model to discriminate between SCDs and mimics of SCD before the CAG strategy in patients who visited the ER complaining of chest pain/discomfort or dyspnea using all relevant clinical data. In addition, we explored how effectively clinical screening by the importance variables deduced from the conventional logistic regression (cLR) method and ML models, can be applied to real-world clinical scenarios.

## Materials and methods

### Study design, setting, and coronary angiography

This multicenter retrospective observational cohort study using data collected in two tertiary teaching hospitals (Dong-A and SamSung Changwon University Hospital) was conducted from November 2013 to August 2020. This study was first approved by the Dong-A University Hospital institutional review board under entry code DAUHIRB-20-050 for internal validation and waived by the SamSung Changwon University Hospital institutional review board for external validation. Informed consent was not required as this study was conducted retrospectively and was waived by the two ethics committee. All methods were performed in accordance with the relevant guidelines and regulations.

**Fig 1:**
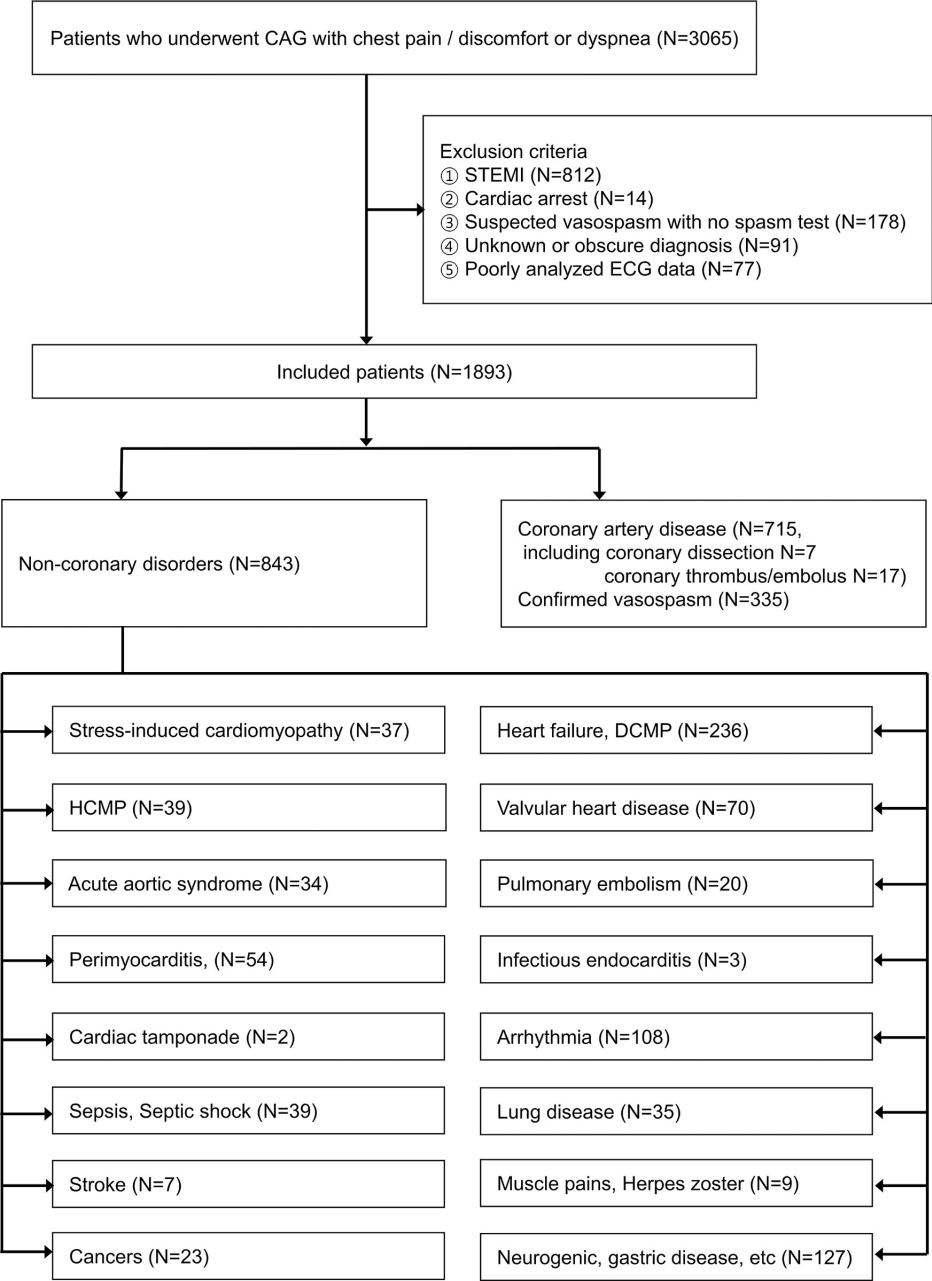
Flowchart.

The factors that enabled us to suspect SCDs at an early stage were investigated (S1 File). First, all clinical data in one of two hospitals were retrieved to conduct conventional analysis to ascertain the correlation of each variable with SCDs and ML for derivation and internal validation. Next, the data sets in the other hospital were used for external validation after identifying the variable importance through ML. As a derivation and internal validation dataset, of 8407 patients who had chest pain/discomfort or dyspnea, 3065 patients in whom CAG was performed were enrolled. We ultimately including 1893 patients, excluding patients with ST elevation myocardial infarction (STEMI) who required immediate treatment; cardiac arrest, which can have diverse causes; uncertain vasospasm that was suspected but for which a spasm test was not performed; an unascertained cause in the final diagnosis; and ECG data that were not analyzed using other ECG devices (Fig 1).

Patients with various diseases that mimic SCDs, including stress-induced cardiomyopathy, heart failure, hypertrophic cardiomyopathy, valvular heart disease, acute aortic syndrome, pulmonary embolism, perimyocarditis, infectious endocarditis, cardiac tamponade, arrhythmia, sepsis or septic shock, lung disease, stroke, muscle pain, herpes zoster, cancers, and neurogenic and gastric diseases, were included and assigned to the non-SCD group (843 patients, Fig 1). Conversely, the patients who underwent percutaneous coronary intervention or ballooning or were diagnosed as SCDs were assigned to the SCD group (1050 patients). Significant CAD was defined as a > 50% reduction in lumen diameter in the left main stem or > 70% stenosis in a major coronary vessel or 30% to 70% stenosis with fractional flow reserve ≤ 0.8 [16], and vasopasm patients who were included in SCDs were confirmed by provocative test.

The importance variables that were significantly correlated with SCDs in the internal validation were extracted for external validation at the other hospital. As a validation dataset, 550 patients at the other hospital were included along the same methods: 372 patients who were diagnosed with CAD, including vasospasm, and 178 patients who belonged to the other disease group. For clinical application, we planned to predict SCDs with the least importance variables, and the most significant variables identified through ML were used for internal and external validation. Additionally, the most significant variables that were used in the fittest model of ML were compared to the importance variables in the model of cLR.

### Data acquisition and definition

All medical findings available when physicians suspected ACS were investigated: demographics and past history; characteristics of chest pain and dyspnea and physical examinations [7]; vital signs and baseline laboratory findings; electrocardiographic and echocardiographic data; and the heart score which consisted of the categories of history (1 point for patients who did not have any clear documentation) [17]. Numerous clinical data were used in our study. The electrocardiographic data were extracted by the converting program (xml to xlsx) that was developed by us and enabled us to use computer-interpreted ECG reports by the Philips 12-Lead algorithm. The ECG variables were revised by defining ST segment depression as the J point in V2 and V3 > 2 mm and > 1 mm in the other leads, T wave inversion as > 1 mm, and pathological Q wave as ≥ 0.03 sec and ≥ 0.1 mV in the QS complex in 2 or more contiguous leads [18].

### Statistical analysis

Fisher’s exact test was used for categorical predictors, and the Mann–Whitney U-test was used for numerical predictors. A cLR was used to determine the association between SCDs and numerous variables. The essential variables to predict SCDs in an univariable analysis were extracted, standardized, and analyzed with KNN imputation for comparing the analytic results in ML methods (S2 File): LR, support vector machine (SVM), random forest; gradient boosting; multi-layer perceptron, extreme gradient boosting (XGBoost), and light gradient boosting machine (lightGBM).

To construct predictive models, data from 1893 patients were divided into training and testing data. In addition, feature-wise normalization was performed because heterogeneous data that all have wildly different ranges would definitely make learning more difficult. Furthermore, 20 different tests as a type of cross-validation were performed by splitting the original data into 20 test cases with random sampling. This procedure avoids overfitting and tunes the model’s hyperparameters during training of the model. For the random forest, the out-of-bag error was applied to assess the performance of the model in addition to cross validation. As a kernel function that quantifies the similarity of two observations in the SVM, a radial kernel function was used. In the boosting, gradient boosting approaches were considered. To select the number of trees in the random forest and boosting, cross-validation was used. Other hyperparameters in addition to the number of trees were tuned based on the out-of-bag error.

Variable importance was obtained by the feature importance with information gain in XGBoost. Then, we reduced the variable dimensionality compatible with the accuracy with the full variables. The missing data in the importance variables that were drawn from the ML methods were replaced by KNN imputation. The importance of variables was validated by analyzing data from the other hospital.

The area under the receiver operating curve (AUROC) to assess the performance of these models was used and plotted using the potential data with or without electrocardiographic and echocardiographic data that were found in the early phase of admission. ML and cLR for internal and external validation were compared. Precision-recall (PRC) curves were constructed to exclude the error of ROC curves that were impacted by the addition of patients without disease but with positive test results.

## Results

Various patients were diagnosed with serious diseases, such as perimyocarditis, acute aortic syndrome or pulmonary embolism, and mild diseases, such as anxiety or gastroesophageal reflux, were included in the control group for comparison with the SCD group. In the univariable analysis, significant variables of all clinical data are shown in Tables 1–3. Forty-six variables were significantly correlated with SCDs (LDH and proBNP, which had 386 and 595 missing data points, respectively, were excluded from subsequent analyses). The heart scores were not different between the SCDs and non-SCD groups (5.16 vs 5.2, p = 0.269).

**Table 1:** Relation between coronary artery disorder and variables in demographic data, history, and physical examination.

Baseline variables	Non-SCD	SCD	*P*
Age, years	66.2 ± 13.88	63.2 ± 11.55	< .001
Male, n (%)	432 (51.2%)	753 (71.7%)	< .001
Body mass index, kg/m^2^	34.4 ± 17.26	36.3 ± 17.34	0.055
Initial systolic blood pressure, mmHg	128.5 ± 21.18	131.8 ±20.04	< .001
Initial diastolic blood pressure, mmHg	75.3 ± 14.42	76.6 ± 12.93	0.024
Initial body temperature, °C	36.4 ± 0.32	36.4 ± 0.26	0.011
Initial heart rate, beats	82 ± 34.13	75.2 ± 15.29	< .001
Initial respiratory rate, times	20 ± 2.79	19.6 ± 1.83	0.001
Initial SpO_2_, %	95.5 ± 3.99	95.9 ± 3.33	0.314
The heart Score	5.2 ± 1.64	5.16 ± 1.66	0.269
** Symptom Character**			
Squeezing pain, n (%)	189 (23%)	403 (41%)	< .001
Pressed pain, n (%)	43 (5.2%)	63 (6.4%)	0.316
Chest discomfort, n (%)	320 (39%)	370 (37.6%)	0.56
Soreness, n (%)	50 (6.1%)	67 (6.8%)	0.566
Tearing pain, n (%)	9 (1.1%)	10 (1%)	1
Burning pain, n (%)	3 (0.4%)	11 (1.1%)	0.104
Stabbing pain, n (%)	32 (3.9%)	39 (4%)	1
Pleuritic pain, n (%)	23 (2.8%)	1 (0.1%)^a^	< .001
Tenderness, n (%)	30 (3.7%)	7 (0.7%)	< .001
Radiating pain, n (%)	65 (7.9%)	115 (11.7%)	0.009
Back pain, n (%)	42 (5.1%)	51 (5.2%)	1
Dyspnea (on exercise), n (%)	410 (50.1%)	272 (27.7%)	< .001
Exertional pain, n (%)	47 (5.7%)	147 (15%)	< .001
Nausea or Vomiting, n (%)	81 (9.9%)	56 (5.8%)	0.001
Diaphoresis, n (%)	64 (7.8%)	89 (9.1%)	0.352
Recent infection^b^, n (%)	97 (11.8%)	40 (4.1%)	< .001
Post-prandial pain, n (%)	12 (1.5%)	6 (0.6%)	0.094
Pitting or pulmonary edema, n (%)	64 (7.8%)	27 (2.7%)	< .001
Emotional stress or tingling sensation, n (%)	21 (2.6%)	24 (2.4%)	0.881
** Past History**			
Hypertension, n (%)	285 (46%)	549 (52.3%)	0.008
Diabetes mellitus, n (%)	177 (28.5%)	330 (31.4%)	0.004
Hypercholesterolemia, n (%)	75 (10.5%)	137 (14%)	0.037
Current smoking, n (%)	197 (23.4%)	309 (29.4%)	0.003
Prior myocardial infarction, n (%)	220 (28%)	398 (41%)	< .001
Prior heart failure, n (%)	96 (12.2%)	43 (4.4%)	< .001
Stroke or brain tumor, n (%)	59 (7.5%)	50 (5.1%)	0.047
Lung disease^c^, n (%)	73 (9.3%)	66 (6.7%)	0.051

^a^ A patient with pleuritic pain was allocated to coronary artery disease group for statistical significance because no patient in non-coronary artery disease group had pleuritic pain.

^b^ Fever, cough, rhinorrhea, and myalgia were included.

^c^ COPD, asthma, lung cancer, and interstitial lung diseases were included.

**Table 2:** Relation between coronary artery disorder and variables in laboratory findings.

Laboratory findings	Non-SCD	SCD	*P*
White blood cell, 10^3^/μL	8.8 ± 3.92	8.1 ± 3.28	< .001
Hemoglobin, g/dL	12.8 ± 2.12	13.5 ± 1.98	< .001
Platelet, 10^3^/μL	231.5 ± 73.16	225.3 ± 65.57	0.054
PT, sec	13.5 ± 4.23	12.5 ± 5.62	< .001
INR	1.2 ± 0.38	1.1 ± 0.48	< .001
D-dimer, μg/mL	2.4 ± 5.64	1.2 ± 2.99	< .001
BUN, mg/dL	20.4 ± 11.54	18.4 ± 9.68	< .001
Creatinine, mg/dL	1.2 ± 0.96	1.2 ± 1.2	0.055
Total cholesterol, mg/dL	168.7 ± 44.95	174 ± 50.8	0.052
Sodium, mmol/L	137.8 ± 4.67	138.7 ± 3.34	< .001
Chloride, mmol/L	103.3 ± 5.16	103.9 ± 3.62	0.05
CK, U/L	200.5 ± 378.27	190.7 ± 424.98	0.43
CK-MB / UNL^a^, times	1 ± 1.21	1.2 ± 2.51	0.083
LDH, U/L	562.9 ± 480.7	469.9 ± 243.1	< .001
CRP, mg/dL	1.9 ± 4.02	1 ± 2.85	< .001
First (hs) Troponin I^b^ / UNL*, times	513.5 ± 7944.74	3397 ± 67732.25	0.004
Second (hs) Troponin I† / UNL*, times	757.5 ± 9695.72	10271.7 ± 224919.66	0.02
Troponin I change per an hour, ng/mL/h	2 ± 38.42	47.1 ± 1208.46	0.121

^a^ Upper normal level (UNL) was defined as the upper reference value supported by assay machine of the parameter.

^b^ Troponin I and high sensitivity troponin I were used as the unit such as pg/mL and ng/mL.

**Table 3:** Relation between coronary artery disorder and variables in electrocardiography and echocardiography.

Variable	Non-SCD	SCD	*P*
**Electrocardiography**			
Rate, times	88.4 ± 32.38	74.1 ± 19.2	< .001
QRS duration, ms	99.7 ± 24.23	97.5 ± 19.07	0.452
QTc, ms	458.2 ± 42.38	440.1 ± 34.23	< .001
Frontal ST axis, °	103.7 ± 97.56	81.1 ± 87.32	< .001
Horizontal ST axis, °	105.7 ± 57.35	100.7 ± 49.27	0.034
Frontal QRS axis, °	38.8 ± 54.52	37.9 ± 45.13	0.583
Horizontal QRS axis, °	3.4 ± 82.12	-6.6 ± 58.18	0.932
Frontal T axis, °	70.9 ± 79.92	55.2 ± 63.67	< .001
Horizontal T axis, °	78.2 ± 61.62	70.6 ± 53.49	0.005
Frontal QRS-T angle, °	186 ± 116.95	181.5 ± 134.72	0.518
Horizontal QRS-T angle, °	230.9 ± 79.39	248.4 ± 79.34	< .001
ST depression*, n (%)	319 (37.8%)	314 (29.9%)	< .001
T inversion^a^, n (%)	327 (38.8%)	304 (29%)	< .001
Pathologic Q*, n (%)	111 (13.2%)	125 (11.9%)	0.441
Minimal ST elevation*, n (%)	181 (21.5%)	295 (28.1%)	0.001
** Echocardiography**			
EF, (%)	51.2 ± 14.65	55.2 ± 10.63	< .001
LVEDD, mm	51.2 ± 8.61	50.5 ± 5.79	0.623
Aortic root diameter, mm	32.6 ± 3.82	32.9 ± 3.49	0.105
LA dimension, mm	39.3 ± 7.59	37.2 ± 5.42	< .001
LA volume, mL	66.7 ± 36.96	55.4 ± 23.24	< .001
E / A ratio	1 ± 0.61	1 ± 0.48	0.04
DT, m/s	200.3 ± 65.19	204.3 ± 51.28	0.007
Mean E/e’	13.9 ± 6.95	11.8 ± 5.49	< .001
Mean s’, cm/s	6.8 ± 1.9	7.4 ± 2	< .001
Mean e’, cm/s	6.2 ± 2.11	6.7 ± 2.02	< .001
RWMA, n (%)	154 (21.1%)	262 (29.1%)	< .001

EF, Ejection fraction; LVEDD, Left ventricular end-diastolic diameter; DT, Deceleration time; RWMA, Regional wall motion abnormality.

^a^ Positive finding of the parameter was observed in contiguous 2 leads or more.

In multivariable analysis via cLR, significant variables were associated with SCDs (Table 4). The most significant variables were regional wall motion abnormality (RWMA) (OR 3.583, p < 0.001), exertional pain (OR 2.844, p < 0.001), male sex (OR 2.194, p < 0.001), squeezing pain (OR 1.861, p < 0.001), prior MI (OR 1.876, p < 0.001), pleuritic pain (OR 0.054, p = 0.002), and chest tenderness (OR 0.357, p = 0.032). The AUC and accuracy were 0.795 and 72.62%, respectively, when SCDs were predicted with the 20 most significant variables. All the data were preprocessed by KNN imputation to be compared to the importance variables in ML.

**Table 4:** Comparison of importance variables in conventional logistic regression and machine learning as multivariable analysis.

Importance variables in conventional LR^a^	Odds Ratio	*P*	95% CI	Importance variables in internal validation^a^	Score
Pleuritic pain	0.054	0.002	0.008─0.344	Tenderness	4.1044
RWMA	3.583	< .001	2.575─4.984	Dyspnea (on exercise)	2.8769
Exertional pain	2.844	< .001	1.931─4.189	RWMA	2.7895
Tenderness	0.357	0.032	0.139─0.916	Exertional pain	2.5739
Male	2.194	< .001	1.725─2.791	Male	2.4069
Dyspnea (on exercise)	0.481	< .001	0.38─0.609	Emotional stress	2.0056
Prior myocardial infarction	1.876	< .001	1.482─2.374	Prior myocardial infarction	1.9647
Squeezing pain	1.861	< .001	1.466─2.363	Squeezing pain	1.9164
Nausea or vomiting	0.622	0.023	0.413─0.936	Recent infection	1.9149
Hypertension	1.475	0.001	1.166─1.867	Nausea or vomiting	1.8849
EF	1.431	< .001	1.225─1.672	Prior heart failure	1.5943
Diabetes mellitus	1.414	0.011	1.085─1.845	Hypertension	1.5034
Heart rate	0.737	< .001	0.621─0.875	Pitting or pulmonary edema	1.4772
LA diameter	0.773	< .001	0.68─0.879	EF	1.4605
QTc	0.786	< .001	0.698─0.886	Respiratory rate	1.4333
CRP	0.805	0.001	0.707─0.916	Age	1.3675
SpO2	0.829	0.001	0.741─0.927	Radiating pain	1.3502
Hemoglobin	1.183	0.008	1.046─1.339	Body mass index	1.3407
Horizontal QRS axis	0.856	0.005	0.767─0.955	QTc	1.2947
Frontal T axis	0.869	0.012	0.778─0.969	LA diameter	1.2662
				SpO2	1.2464

^a^ Continuous variables on a different level were standardized for analysis.

KNN imputation was used as the same method for all missing data to be analyzed by conventional logistic regression and machine learning.

**Fig 2:**
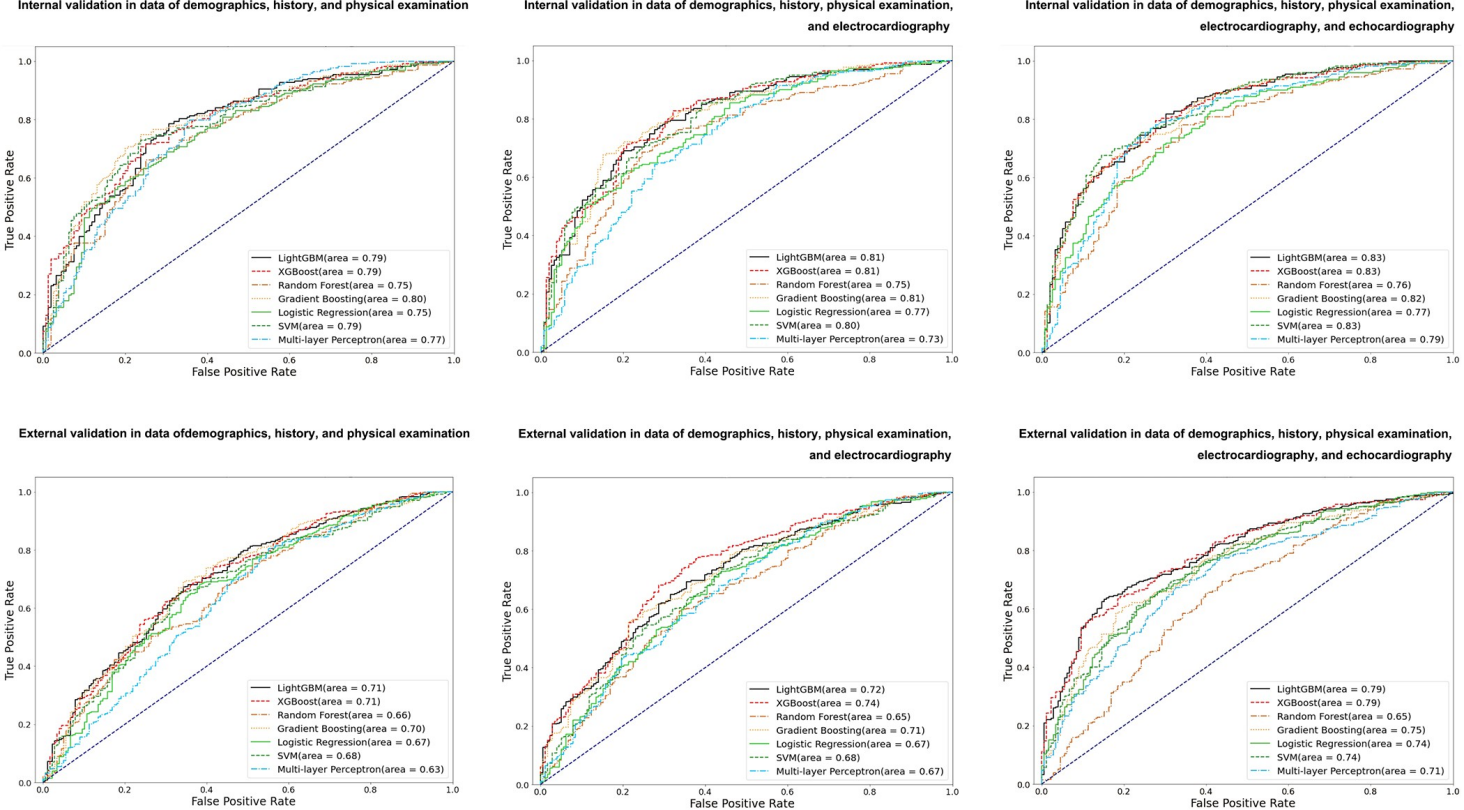
Internal and external validation according to dataset. Left column: Performance using demographic, history, and physical examination data. Middle column: Performance adding electrocardiographic data. Right column: Performance adding electrocardiographic and echocardiographic data. Area indicates the area under the receiver operating characteristic curve; lightGBM, light gradient boosting machine; XGBoost, extreme gradient boosting: SVM, support vector machine.

**Fig 3:**
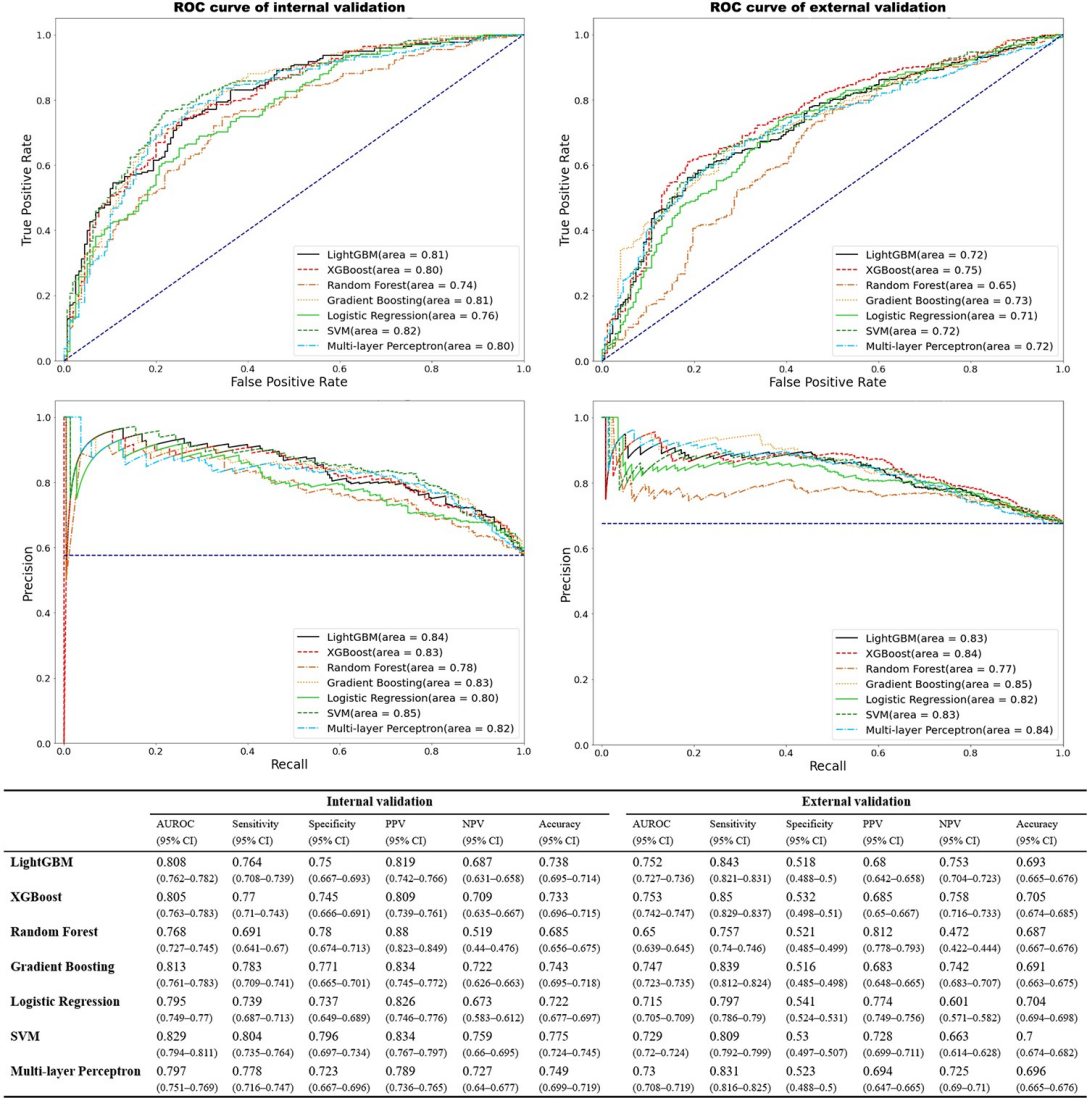
The precision using the fittest model with the importance variables determined by machine learning.

In multivariable analysis via ML, the AUCs in the internal validation were 0.8 with demographic, history, and physical examination data; 0.81 with the preceding data plus electrographic data; and 0.83 with the preceding data plus echocardiographic data, and in external validation, the AUCs were 0.71 with demographic, history, and physical examination data; 0.74 with the preceding data plus electrographic data; and 0.79 with the preceding data plus echocardiographic data (Fig 2). The accuracy of the analysis using ML with all 85 variables was 74.98%. The 44 importance variables extracted by internal validation were tested to prove the external validation. The 21 fittest variables sifted through for clinical application were compared to the significant variables in conventional multivariable LR (Table 4). The AUCs and accuracy of the fittest model were 0.81 and 72.48% in the internal validation and 0.75 and 70.5% in the external validation, respectively (Fig 3). The most significant variables in the fittest model were chest tenderness, dyspnea on exercise, squeezing pain, exertional pain, recent infection, RWMA, prior heart failure, prior MI, and nausea or vomiting in a sequence (Table 4).

Electrocardiographic variables had little ability to predict SCDs in all analytic methods. The LightGBM and XGBoost analyses including all significant variables (85 and 46 variables in the internal and external validation datasets, respectively) were the best methods to predict SCDs (AUC 0.83 and 0.79 in the internal and external validation datasets, respectively). The fittest model using 21 variables was a competitive discriminator, as was the model using 85 variables. The levels of predictive power of ML and cLR were not largely different, although the composition of the fittest variables showed small differences in each analysis.

## Discussion

Non-SCDs was best distinguishable from SCDs when using ML with 85 variables, but it showed no major difference with the fittest model using 21 variables. Additionally, the predictive powers of cLR and ML were also not largely different. Common importance variables from cLR and ML were RWMA, exertional pain, tenderness, male sex, dyspnea (on exercise), prior myocardial infarction, squeezing pain, nausea or vomiting, hypertension, ejection fraction, left atrial diameter, and QTc. Moreover, pleuritic pain, emotional stress, recent infection, and diabetes mellitus were likely to deserve consideration as significant variables, because the importance of variables can vary according to the distribution of patients included in the non-SCD group. The models determined to be predictive by ML were proven to be an available tool to predict significant SCDs at an early clinical stage in patients suspected of having non-ST elevation ACS. To the best of our knowledge, there were few studies that incorporated patients with vasospasm differentiating acute coronary syndrome from severe mimicking diseases as predictive analytic modeling to consider a coronary angiography. Those importance variables would be used to distinguish acute coronary syndrome from various mimicking diseases at an early stage.

In previous studies, stabbing, pleuritic chest pain, and reproducible chest wall pain on palpation were less associated with ACS (likelihood ratios 0.2–0.3), and radiating and exertional chest pain were most associated with ACS (likelihood ratios 2.3–4.7) [7]. Pressed chest pain, nausea or vomiting, diaphoresis, and prior MI were probable risks [7,19]. Pressed chest pain and diaphoresis were not significant variables, nausea or vomiting was more related to non-SCDs, and radiating pain was not a considerable predictor in our study. These differences may result from incorporating a small number of non-SCD patients with questionable chest discomfort and dyspnea in the studies. Another study showed that nausea, vomiting, and diaphoresis showed no relation to CAD [20].

Demographic findings, such as age and male sex, and past history, such as diabetes mellitus, current smoking, dyslipidemia, and previous MI other than hypertension were good predictors of CAD [16,21], while hypertension was a good predictor in other studies [22,23]. Systolic blood pressure, diastolic blood pressure, and heart rate were not related to CAD [23], but blood pressure was a predictor of CAD in another study [22]. Palpitation had an inverse relation with CAD [19]. Most conventional risk factors were correlated with SCDs in our results, but the mean blood pressure was not substantially different between the two groups. Sinus tachycardia or tachyarrhythmia due to a severe illness might be associated with non-CAD, as in our results.

The heart score as the TIMI or GRACE score is a reliable factor that predicts major adverse cardiac events [24], and it showed higher predictive power than the TIMI or GRACE score in ACS risk stratification [25]. The heart score as a prognostic factor was not included in the analyses to predict CAD. The risk level showing the heart score in non-SCDs was identical to that in SCDs. Initial troponin I as a component of the heart score was not associated with SCDs. Steep elevation of troponin I at an early phase might not be presented in patients with chronic chest symptom, a bunch of collateral vessels, or vasospasm. Conversely, severe mimicking diseases, such as perimyocarditis, septic shock, pulmonary embolism, and valvular heart disease, might increase the level of troponin I. Our study included many patients with mimicking diseases at considerable risk and with vasospasm and the factors may affect the initial level of troponin I. Moreover, abnormality in ST segment and T wave as another component of the heart score showed no significant correlation with SCDs. In ML, ST depression and T inversion have been used as variables to distinguish CAD [11,15]. ST depression and T inversion are strong prognostic factors [26], but not a good diagnostic tool. Compared with the characteristics of chest pain, they were not good discriminators in terms of diagnosing CAD [27]. The diagnostic significance of ST depression and T inversion was also not revealed in either cLR or ML in our multivariable analysis; rather, variables such as ventricular rate, QTc, horizontal QRS axis, and frontal T axis were more related to CAD.

A total of 52.8% of patients with CAD among the NSTEMI patients visiting the ER had RWMA, but the RWMA was also shown in 43.7% of patients with non-CAD and RWMA failed to distinguish between CAD and non-CAD with no difference in peak troponin T in a previous study [28]. RWMA cannot be found in all NSTEMI patients, and it can also be observed in patients with troponin-positive nonobstructed coronary arteries [29]. If the number of patients with mimicking diseases are included in studies, RWMA may become less effective as a discriminator of SCDs. RWMA in our study was a significant predictor. How mild mimicking diseases were included may be a vital clue to give importance to RWMA.

Distinguishing SCDs and non-SCDs using ML can reduce physician fatigue and the number of misdiagnosed cases. In cases using simple history taking and basic information, ML showed an AUC of 0.76–0.8 [8,14]. Using more variables, such as demographic data, past history, symptom characteristics, physical examination, electrocardiographic data, and echocardiographic data, ML showed higher diagnostic power with an AUC of 0.93 [13,15]. However, these recent studies did not include troponin I, severe non-SCDs, full datasets regarding chest symptoms and past history, nor did they conduct an external validation. Furthermore, distinguishing SCDs, including vasospasm, from non-SCDs, including severe mimics, can be essential to apply to various clinical scenarios where physicians practically suspect CAD and consider performing CAG.

## Limitations

This study has several limitations. First, this was a retrospective observational study conducted in two hospitals. There is a possibility of overfitting in this case, but we minimized this possibility statistically by using a random sampling method for cross-validation and out-of-bag error. Second, several diseases, such as diseases of unknown cause and suspected vasospasm without spasm tests were excluded. These obscure diseases must be classified as more specific diseases. Third, the quantity of mimics with a severe illness in the non-SCD group might change the importance of variables when predicting SCDs with our algorithm in patients with chest symptoms. Last, the predictive ability of the models was not much high. Which may be because a number of patients with vasospasm and severe mimicking diseases that may not be plainly distinguishable were included in our study. Previously, there have not been studies on prediction of SCDs including vasospasm and predicting SCDs excluding many vasospasm patients may be not available for consideration of CAG in various clinical scenario.

## Conclusion

SCDs were significantly predicted through ML models using all clinical data that were collected at an early stage of admission from the patients suspected of having non-ST elevation ACS. The analysis including 21 significant variables to predict SCDs was not largely different from the analysis using all 85 variables. The variables with high-ranking importance that were selected in the fittest model may be promising discriminators in various clinical scenarios in which ACS is suspected and should be screened to differentiate the mimicking diseases for which coronary angiography is not required. Screening these importance variables in patients suspected of having ACS at an early stage of admission can assist physicians in distinguishing SCDs from mimicking diseases.

## Supporting information

S1 File Investigated variables. (DOCX)

S2 File Minimal underlying data set. (XLSX)
