# Supplementary material for: Prediction of disorders with significant coronary lesions using machine learning in patients admitted with chest symptom
Source: PLoS One. 2022 Oct 10;17(10):e0274416. doi: 10.1371/journal.pone.0274416 (PMC9550076; doi:10.1371/journal.pone.0274416)
Supplement: S1 File — (DOCX) [file pone.0274416.s001.docx]

**Investigated variables**

**Dependent variable :** final diagnosis, CAD or not

**Demographics :** age, sex, height, weight, BMI, Obesity(25kg/m2),

**Symptom characteristics :** squeezing pain, pressed pain, chest discomfort, chest soreness, tearing, burning, pricky pain, pleuritic pain, chest wall pain, epigastric tenderness, dyspnea or DOE, excertional chest pain, radiating pain, sweating, nausea or vomiting, postprandial pain, back pain, recent infection (cough or rhinorrhea or myalgia), pitting edema, pulmonary edema, edema, tingling sensation, HT, DM, Dyslipidemia, current_smoking, PHx_Angina, CAD, MI, PHx_IHD, Family history, PHx_CHF, PHx_stroke or brain mass, PHx_lung Ds (COPD, asthma, lung ca, ILD),

**Physical exam and laboratory finding :** SBP, DBP, BT, HR, RR, SPO2, WBC, RBC, Hb, HEMATOCRIT, PLT, Seg_neutrophil, Seg_lymphocyte, NLR, ESR, PT, INR, APTT, D-dimer, Total_Ca, Phosphorus, glucose, BUN, Creatinine, BUN_Cr ratio, GFR, uric acid, Total_Cholesterol, Total protein, Albumin, AST, ALT, ALP, T_Bil, D_Bil, GGT, Na, K, Cl, Amylase, Lipase, CK, CKMB, CKMB_UNL, LDH, Osm_serum, ketone, CRP, First_TnI_time, First TnI_(ng), Troponin_I_UNL (ng/mL), Second_TnI_time, Second_TnI_(ng), delta_TnI_hr, ProBNP, ProBNP_UNL, Lactate, urine_Na, Urine Ph, urine_Protein, urine_Glucose, urine_Ketone, urine_Bilirubin, urine_RBC (HPF), urine_WBC_(HPF), PH, PCO2, PO2, Bicarbonate, TCO2,

**Echo :** RWMA(+), EF, LVIDD, LVIDS, LVSD, LVPWD, LVOTD, TVI, AORTIC_ROOT, ASCENDING_AORTA, LA_DIMENSION, LA_VOLUME, RA, SEPTAL_Sa, SEPTAL_Ea, SEPTAL_Aa, LATERAL_Sa, LATERAL_Ea, LATERAL_Aa, SEPTAL_E_Ea, LATERAL_E_Ea, Mean_E_Ea, Mean_Sa, Mean_Ea, LV_RELAXATION, LA_PRESSURE, RA_PRESSURE, STROKE_VOLUME, HEART_RATE, AV_PEAK_VELOCITY, AV_MEAN_GRADIENT, AV_TVI, LVOT_TVI, MITRAL_E, MITRAL_A, E_A_RATIO, DT,

**ECG :** pfrontaxis, phorizaxis, qrsfrontaxis, absolute_qrs_frontal_axis, qrshorizaxis, absolute_qrs_horizontal_axis, stfrontaxis, absolute_st_frontal_axis, sthorizaxis, absolute_st_horizontal_axis, tfrontaxis, absolute_t_frontal_axis, thorizaxis, absolute_t_horizontal_axis, frontal_QRS_T_axis, horizontal_QRS_T_axis, atrialrate, meanventrate, meanprint, meanprseg, meanqrsdur, meanqtint, meanqtc, transpmaxmag, transqrsmaxmag, transtmaxmag, frontpmaxmag, frontqrsmaxmag, fronttmaxmag, sagpmaxmag, sagqrsmaxmag, sagtmaxmag, I_qamp (Q AMP)100, I_qamp (Q AMP), I_qdur (Q DUR), I_Pathologic_Q, I_vat (V.A.T.), I_qrsppk (QRS PPK)100, I_qrsppk (QRS PPK), I_qrsdur (QRS DUR), I_ston (ST ON)100, I_ston (ST ON), I_STD, I_stmid (ST MID), I_st80 (ST 80ms)100, I_st80 (ST 80ms), I_STD80, I_stend (ST END), I_stslope (STSLOPE), I_Stslope(up sloping, horizontal, down sloping), I_stshape (STSHAPE), I_tamp (T AMP)100, I_tamp (T AMP), I_tpamp (T' AMP)100, I_tpamp (T' AMP), I_T_min(inversion), I_T_inversion, I_T_amplitude, II_qamp (Q AMP)100, II_qamp (Q AMP), II_qdur (Q DUR), II_Pathologic_Q, II_vat (V.A.T.), II_qrsppk (QRS PPK)100, II_qrsppk (QRS PPK), II_qrsdur (QRS DUR), II_ston (ST ON)100, II_ston (ST ON), II_STD, II_stmid (ST MID), II_st80 (ST 80ms)100, II_st80 (ST 80ms), II_STD80, II_stend (ST END), II_stslope (STSLOPE), II_Stslope(up sloping, horizontal, down sloping), II_stshape (STSHAPE), II_tamp (T AMP)100, II_tamp (T AMP), II_tpamp (T' AMP)100, II_tpamp (T' AMP), II_T_min(inversion), II_T_inversion, II_T_amplitude, III_qamp (Q AMP)100, III_qamp (Q AMP), III_qdur (Q DUR), III_Pathologic_Q, III_vat (V.A.T.), III_qrsppk (QRS PPK)100, III_qrsppk (QRS PPK), III_qrsdur (QRS DUR), III_ston (ST ON)100, III_ston (ST ON), III_STD, III_stmid (ST MID), III_st80 (ST 80ms)100, III_st80 (ST 80ms), III_STD80, III_stend (ST END), III_stslope (STSLOPE), III_Stslope(up sloping, horizontal, down sloping), III_stshape (STSHAPE), III_tamp (T AMP)100, III_tamp (T AMP), III_tpamp (T' AMP)100, III_tpamp (T' AMP), III_T_min(inversion), III_T_inversion, III_T_amplitude, aVR_qamp (Q AMP)100, aVR_qamp (Q AMP), aVR_qdur (Q DUR), aVR_Pathologic_Q, aVR_vat (V.A.T.), aVR_qrsppk (QRS PPK)100, aVR_qrsppk (QRS PPK), aVR_qrsdur (QRS DUR), aVR_ston (ST ON)100, aVR_ston (ST ON), aVR_STD, aVR_stmid (ST MID), aVR_st80 (ST 80ms)100, aVR_st80 (ST 80ms), aVR_STD80, aVR_stend (ST END), aVR_stslope (STSLOPE), aVR_Stslope(up sloping, horizontal, down sloping), aVR_stshape (STSHAPE), aVR_tamp (T AMP)100, aVR_tamp (T AMP), aVR_tpamp (T' AMP)100, aVR_tpamp (T' AMP), aVR_T_min(inversion), aVR_T_inversion, aVR_T_amplitude, aVL_qamp (Q AMP)100, aVL_qamp (Q AMP), aVL_qdur (Q DUR), aVL_Pathologic_Q, aVL_vat (V.A.T.), aVL_qrsppk (QRS PPK)100, aVL_qrsppk (QRS PPK), aVL_qrsdur (QRS DUR), aVL_ston (ST ON)100, aVL_ston (ST ON), aVL_STD, aVL_stmid (ST MID), aVL_st80 (ST 80ms)100, aVL_st80 (ST 80ms), aVL_STD80, aVL_stend (ST END), aVL_stslope (STSLOPE), aVL_Stslope(up sloping, horizontal, down sloping), aVL_stshape (STSHAPE), aVL_tamp (T AMP)100, aVL_tamp (T AMP), aVL_tpamp (T' AMP)100, aVL_tpamp (T' AMP), aVL_T_min(inversion), aVL_T_inversion, aVL_T_amplitude, aVF_qamp (Q AMP)100, aVF_qamp (Q AMP), aVF_qdur (Q DUR), aVF_Pathologic_Q, aVF_vat (V.A.T.), aVF_qrsppk (QRS PPK)100, aVF_qrsppk (QRS PPK), aVF_qrsdur (QRS DUR), aVF_ston (ST ON)100, aVF_ston (ST ON), aVF_STD, aVF_stmid (ST MID), aVF_st80 (ST 80ms)100, aVF_st80 (ST 80ms), aVF_STD80, aVF_stend (ST END), aVF_stslope (STSLOPE), aVF_Stslope(up sloping, horizontal, down sloping), aVF_stshape (STSHAPE), aVF_tamp (T AMP)100, aVF_tamp (T AMP), aVF_tpamp (T' AMP)100, aVF_tpamp (T' AMP), aVF_T_min(inversion), aVF_T_inversion, aVF_T_amplitude, V1_qamp (Q AMP)100, V1_qamp (Q AMP), V1_qdur (Q DUR), V1_Pathologic_Q, V1_vat (V.A.T.), V1_qrsppk (QRS PPK)100, V1_qrsppk (QRS PPK), V1_qrsdur (QRS DUR), V1_ston (ST ON)100, V1_ston (ST ON), V1_STD, V1_stmid (ST MID), V1_st80 (ST 80ms)100, V1_st80 (ST 80ms), V1_STD80, V1_stend (ST END), V1_stslope (STSLOPE), V1_Stslope(up sloping, horizontal, down sloping), V1_stshape (STSHAPE), V1_tamp (T AMP)100, V1_tamp (T AMP), V1_tpamp (T' AMP)100, V1_tpamp (T' AMP), V1_T_min(inversion), V1_T_inversion, V1_T_amplitude, V2_qamp (Q AMP)100, V2_qamp (Q AMP), V2_qdur (Q DUR), V2_Pathologic_Q, V2_vat (V.A.T.), V2_qrsppk (QRS PPK)100, V2_qrsppk (QRS PPK), V2_qrsdur (QRS DUR), V2_ston (ST ON)100, V2_ston (ST ON), V2_STD, V2_stmid (ST MID), V2_st80 (ST 80ms)100, V2_st80 (ST 80ms), V2_STD80, V2_stend (ST END), V2_stslope (STSLOPE), V2_Stslope(up sloping, horizontal, down sloping), V2_stshape (STSHAPE), V2_tamp (T AMP)100, V2_tamp (T AMP), V2_tpamp (T' AMP)100, V2_tpamp (T' AMP), V2_T_min(inversion), V2_T_inversion, V2_T_amplitude, V3_qamp (Q AMP)100, V3_qamp (Q AMP), V3_qdur (Q DUR), V3_Pathologic_Q, V3_vat (V.A.T.), V3_qrsppk (QRS PPK)100, V3_qrsppk (QRS PPK), V3_qrsdur (QRS DUR), V3_ston (ST ON)100, V3_ston (ST ON), V3_STD, V3_stmid (ST MID), V3_st80 (ST 80ms)100, V3_st80 (ST 80ms), V3_STD80, V3_stend (ST END), V3_stslope (STSLOPE), V3_Stslope(up sloping, horizontal, down sloping), V3_stshape (STSHAPE), V3_tamp (T AMP)100, V3_tamp (T AMP), V3_tpamp (T' AMP)100, V3_tpamp (T' AMP), V3_T_min(inversion), V3_T_inversion, V3_T_amplitude, V4_qamp (Q AMP)100, V4_qamp (Q AMP), V4_qdur (Q DUR), V4_Pathologic_Q, V4_vat (V.A.T.), V4_qrsppk (QRS PPK)100, V4_qrsppk (QRS PPK), V4_qrsdur (QRS DUR), V4_ston (ST ON)100, V4_ston (ST ON), V4_STD, V4_stmid (ST MID), V4_st80 (ST 80ms)100, V4_st80 (ST 80ms), V4_STD80, V4_stend (ST END), V4_stslope (STSLOPE), V4_Stslope(up sloping, horizontal, down sloping), V4_stshape (STSHAPE), V4_tamp (T AMP)100, V4_tamp (T AMP), V4_tpamp (T' AMP)100, V4_tpamp (T' AMP), V4_T_min(inversion), V4_T_inversion, V4_T_amplitude, V5_qamp (Q AMP)100, V5_qamp (Q AMP), V5_qdur (Q DUR), V5_Pathologic_Q, V5_vat (V.A.T.), V5_qrsppk (QRS PPK)100, V5_qrsppk (QRS PPK), V5_qrsdur (QRS DUR), V5_ston (ST ON)100, V5_ston (ST ON), V5_STD, V5_stmid (ST MID), V5_st80 (ST 80ms)100, V5_st80 (ST 80ms), V5_STD80, V5_stend (ST END), V5_stslope (STSLOPE), V5_Stslope(up sloping, horizontal, down sloping), V5_stshape (STSHAPE), V5_tamp (T AMP)100, V5_tamp (T AMP), V5_tpamp (T' AMP)100, V5_tpamp (T' AMP), V5_T_min(inversion), V5_T_inversion, V5_T_amplitude, V6_qamp (Q AMP)100, V6_qamp (Q AMP), V6_qdur (Q DUR), V6_Pathologic_Q, V6_vat (V.A.T.), V6_qrsppk (QRS PPK)100, V6_qrsppk (QRS PPK), V6_qrsdur (QRS DUR), V6_ston (ST ON)100, V6_ston (ST ON), V6_STD, V6_stmid (ST MID), V6_st80 (ST 80ms)100, V6_st80 (ST 80ms), V6_STD80, V6_stend (ST END), V6_stslope (STSLOPE), V6_Stslope(up sloping, horizontal, down sloping), V6_stshape (STSHAPE), V6_tamp (T AMP)100, V6_tamp (T AMP), V6_tpamp (T' AMP)100, V6_tpamp (T' AMP), V6_T_min(inversion), V6_T_inversion, V6_T_amplitude, Ant_Tmax_c2leads, Ant_QRSmax_c2leads, Ant_TQRSmax_c2leads, Ant_Tmin_c2leads, Ant_QRSmin_c2leads, Ant_TQRSmin_c2leads, Ant_STD_c2leads, Ant_ST_upsloping, Ant_TI_c2leads, Ant_pQ_c2leads, Inf_Tmax_c2leads, Inf_QRSmax_c2leads, Inf_TQRSmax_c2leads, Inf_Tmin_c2leads, Inf_QRSmin_c2leads, Inf_TQRSmin_c2leads, Inf_STD_c2leads, Inf_ST_upsloping, Inf_TI_c2leads, Inf_pQ_c2leads, Lat_Tmax_c2leads, Lat_QRSmax_c2leads, Lat_TQRSmax_c2leads, Lat_Tmin_c2leads, Lat_QRSmin_c2leads, Lat_TQRSmin_c2leads, Lat_STD_c2leads, Lat_ST_upsloping, Lat_TI_c2leads, Lat_pQ_c2leads, TQRSmax, TQRSmin, STD, TI, pQ, Ant_STE_c2leads, Ant_miniSTE_c2leads, Inf_STE_c2leads, Inf_miniSTE_c2leads, Lat_STE_c2leads, Lat_miniSTE_c2leads, STE, mSTE,

**The_Heart_Score.**
